# Single or double endoloop ligation in laparoscopic appendicectomy: a mixed-methods study of clinical outcomes and surgeon perspectives

**DOI:** 10.1186/s12893-026-03544-5

**Published:** 2026-02-02

**Authors:** Lara Nassar, Miqdad Qandeel, Philobater Awad, Basma Hassan, Lina Alim, Jimena Alvarez Del Castillo Gonzalez, Mustafa Makkiyah, Jasim Al-Musawi

**Affiliations:** 1 Emergency General Surgery Unit, Northwick Park Hospital, London North West University Healthcare NHS Trust, London, UK; 2 General and Breast Surgery Department, West Middlesex University Hospital, Chelsea and Westminster Trust, London, UK; 3 Colorectal Surgery, The Royal Marsden Hospital, London, UK

**Keywords:** Laparoscopic appendicectomy, Endoloop, Appendiceal stump closure, One endoloop versus two endoloops, Single endoloop versus double endoloop, Post-operative complications, Mixed-methods, Operative time, Focus group

## Abstract

**Background:**

Securing the appendiceal stump is a critical step in laparoscopic appendicectomy, the gold-standard treatment for managing acute appendicitis. Endoloops are widely used in appendiceal stump ligation owing to their simplicity and cost-effectiveness, but practice varies between surgeons in the use of one or two loops. This study aimed to compare outcomes between single and double endoloop closure and to explore surgeon perspectives.

**Methods:**

A mixed methods approach was used to compare the use of one or two endoloops. A retrospective cohort analysis included patients undergoing laparoscopic appendicectomy in a UK district general hospital between August 2023 and January 2025, comparing post-operative complications and operating time between single and double endoloop ligation. Overall complications were defined as any complications within the 30-day post-operative period while clinically relevant complications referred to intra-abdominal abscess, stump leak, or stump appendicitis. A focus group of operating surgeons was analysed thematically to explore decision-making factors.

**Results:**

Among 191 patients included, 50 (26%) received a single endoloop and 141 (74%) received two. No statistically significant difference in clinically relevant 30-day complications was observed (12% vs. 9.9%; *p* = 0.07) with overlapping confidence intervals for all complication outcomes. Operative time was shorter in the single-endoloop group (84 ± 41.5 vs. 108.5 ± 31.9 min; *p* = 0.004). Thematic analysis identified three key influences on endoloop choice: perceived security through tradition, assessment of appendiceal base integrity, and training considerations.

**Conclusion:**

No statistically significant difference in clinically relevant post-operative complications was observed between single and double endoloop closure, although the study may be underpowered to detect rare events. Single endoloop closure was associated with a shorter operative time, which may be partly confounded by disease severity. Surgical decision-making remains influenced by training culture and tradition rather than evidence-based practice, and full standardisation is challenging given variability in case complexity and surgeon expertise.

**Supplementary Information:**

The online version contains supplementary material available at 10.1186/s12893-026-03544-5.

## Background

Acute appendicitis is a common surgical emergency, and laparoscopic appendicectomy (LA) is the gold-standard operative approach in most centres due to reduced post-operative pain, length of stay and surgical site infection (SSI) rates compared with open surgery [1–3]. A key operative step is secure closure of the appendiceal stump [4, 5]. Various laparoscopic techniques are used to achieve this, including pre-knotted surgical ties (endoloops), intra-corporeal sutures, stapling devices, clipping devices and electrothermal instruments [2]. However, no technique has demonstrated clear superiority in terms of post-operative complications, resulting in marked variation in surgical practice [2, 4–6].

Although LA has been associated with higher rates of intra-abdominal collections compared with open surgery [3, 4], these complications remain uncommon and are influenced primarily by disease severity, contamination, and local tissue condition. Nonetheless, concerns about a potential association with stump leak persist [4, 5]. In contemporary practice, appendiceal stump closure therefore emphasises operative efficiency, cost, and appropriate technique selection based on appendiceal stump integrity, rather than pursuit of a universally optimal technique. Endoloops are widely used because they are simple, readily available, and cost-effective, with clinical outcomes comparable to mechanical devices, although the latter may shorten operating time at higher per-case cost. Within endoloop use, variation exists in whether surgeons secure the stump with one or two ligatures [2–4, 6–8]. Existing retrospective series and limited trial data generally show no difference in post-operative complications between single and double loop ligation, although single-loop use may reduce operative time and consumable costs [5, 9–11]. These findings are frequently confounded by case severity, tissue friability and trainee involvement, contributing to persistent heterogeneity in practice [2].

To our knowledge, this is the first UK-based mixed-methods study integrating clinical outcome data with surgeon reasoning to explain persistent variation in appendiceal stump endoloop closure. While previous studies have reported overall post-operative complication rates, few have focused specifically on clinically relevant stump-related complications such as intra-abdominal abscess, stump leak or stump appendicitis. Our aims were to compare clinically relevant outcomes and operative time between single and double endoloop ligation in LA and explore factors influencing surgeon decision-making.

## Patients and methods

### Study design

A mixed-methods study was conducted, comprising a retrospective cohort analysis and a qualitative focus group. The study was undertaken in the Emergency General Surgery Unit of a UK district general hospital.

### Quantitative element

All patients undergoing laparoscopic appendicectomy between August 2023 and January 2025 were identified from a prospectively maintained theatre database. This timeframe was selected following implementation of a unified electronic health record system in August 2023, ensuring complete and standardised clinical documentation.

Cases were excluded if there was conversion to open surgery, if the appendiceal stump was secured using a method other than endoloops, or if operative documentation was incomplete. Patients were categorised into single endoloop (1EL) and double endoloop (2EL) groups, according to the number of endoloops applied, and outcomes were compared between these groups. Data extraction included age, sex, ASA grade, operative duration, intra-operative severity (perforation, gangrene, and appendiceal base integrity), histology and 30-day postoperative outcomes. Clinically relevant stump-related complications were defined a priori as intra-abdominal abscess, stump leak, or stump appendicitis. Complications were graded using Clavien-Dindo classification (CD) [12].

### Statistical analysis

Data were analysed using IBM SPSS Statistics version 26 (IBM Corp., Armonk, NY, USA). Continuous variables were reported as mean ± standard deviation and compared using the independent t-test. Categorical variables were compared using Chi-squared test or Fishers exact test where appropriate. A *p*-value < 0.05 was considered statistically significant. Confidence intervals were calculated for key outcome measures, including operative time and complication rates. Adjustment for disease severity was not feasible due to dataset constraints and is acknowledged as a limitation.

### Operative technique

All laparoscopic appendicectomies were performed under general anaesthesia by the Emergency General Surgery team at Northwick Park Hospital. Procedures were primarily trainee-led with consultant supervision, reflecting routine practice within the unit. Consultants directly operated in selected complex cases. Endoloops were passed around the base of the appendix and tightened to securely ligate the stump following appendiceal and mesoappendix dissection. The decision to apply one versus two endoloops was at the operating surgeon’s discretion and often based on intra-operative assessment of appendiceal base integrity. In all cases, the stump was ligated with one or more endoloops, and the appendix was divided and retrieved via a 10-mm port.

### Qualitative element

A focus group of eight surgeons (three consultants, five trainees) who regularly perform LA was conducted to explore factors influencing stump ligation choice. The chief investigator of the study, a consultant general surgeon, chaired the session using a semi-structured format with predetermined questions to guide discussion (see Appendix 3). The facilitator did not contribute personal opinions during the discussion in order to minimise influence on participant responses. The session was conducted via Microsoft Teams, recorded, transcribed verbatim, and anonymised.

The transcript was coded in NVivo 12 (QSR International) and analysed using inductive thematic analysis following Braun and Clarke [13]. Coding was performed by one investigator and independently reviewed by a second, with discrepancies resolved through discussion. Participant quotations are presented using role-based identifiers to preserve anonymity while providing contextual clarity. Data were analysed until no new concepts emerged, suggesting thematic saturation.

### Ethical considerations

The study received Health Research Authority (HRA) and Health and Care Research Wales (HCRW) approval through the Integrated Research Application System (IRAS project ID: 355719; REC reference: 25/PR/0988). In accordance with the HRA and HCRW decision, formal Research Ethics Committee (REC) approval was not required for this study. Local capacity and capability were confirmed by the participating NHS Trust prior to study initiation. The qualitative component involved surgeons only, with written informed consent obtained for participation, audio recording, and the use of anonymous quotations in the manuscript. The study was conducted in accordance with the principles of the Declaration of Helsinki.

## Results

### Quantitative findings

#### Study cohort

**Fig. 1:**
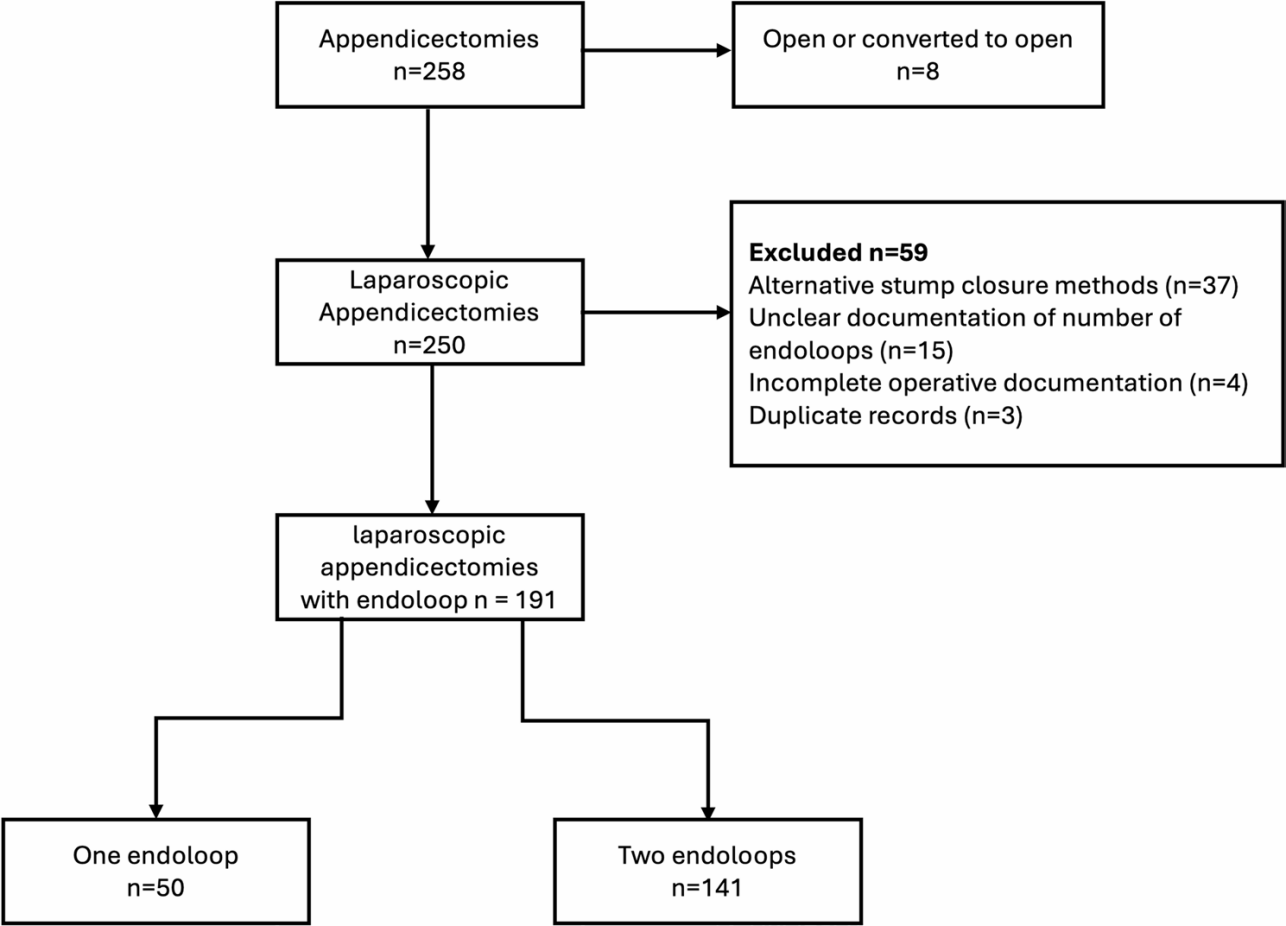
Flow diagram of patient selection

A total of 258 appendicectomies were performed during the study period. Eight were open or converted to open, and 250 were completed laparoscopically. Of these, 37 used alternative stump closure methods, 15 had unclear documentation of the number of endoloops, four had incomplete operative documentation and three were duplicate records. A total of 191 laparoscopic appendicectomies with endoloop stump ligation were included: 141 (74%) using 2EL and 50 (26%) using 1EL (Fig. 1).

#### Baseline characteristics

Demographic and baseline characteristics were similar between groups. Mean age was 35.2 ± 6.2 years in the 2EL group and 39.5 ± 5.5 years in the 1EL group. Overall, 55% were male and mean ASA grade was 1.5 ± 0.6.

Intra-Operative Findings and Operative Duration.

Perforated appendicitis was found in 46 patients (24%): 28/141 (19.9%) in the 2EL group and 18/50 (36%) in the 1EL group (*p* = 0.06). An unhealthy or gangrenous appendiceal base was documented in six patients (3%), evenly distributed across groups.

Mean operative time was shorter in the 1EL group (84.04 ± 41.5 min) compared with the 2EL group (108.54 ± 31.9), with a mean difference of 24.5 min *p* = 0.004) (Table 1).

**Table 1:** Summary of intra-operative findings

Parameter	Double Endoloop	Single Endoloop	*p*-value
Mean operative time (min ± SD, CI)	108.54 ± 31.9, 95% CI 103.3-113.8	84.04 ± 41.5, 95% CI 72.5–95.6	0.004
Perforated appendix (n (%))	28 (19.9%)	18 (36%)	0.06
Gangrenous base (n (%))	3 (6%)	3 (6%)	0.64

Continuous variables are presented as mean ± SD with 95% CI, and categorical variables as number (percentage)

#### Post-operative outcomes

Overall 30-day post-operative complications occurred in 28/191 patients (14.7%): 20/141 (14.1%) in the 2EL group and 8/50 (16.0%) in the 1EL group with no statistically significant difference detected (*p* = 0.92). Of those, 21 complications were a Clavien-Dindo grade 2 while seven were a grade 3a. Clinically relevant stump-related complications occurred in 16 patients (8.4% of the total cohort), comprising 15 intra-abdominal collections and one case of stump appendicitis. Six patients required image-guided drainage. No stump leaks or mortality were observed. Twelve patients (6.3%) had complications unrelated to the appendiceal stump such as pulmonary embolism, acute respiratory distress syndrome, incisional hernia and seroma. Relevant stump-related complications occurred in 14/141 patients (9.9%) in the 2EL and 6/50 patients (12.0%) in the 1EL group (*p* = 0.07). Although not statistically significant, this difference was directionally higher in the single-endoloop group. In a sensitivity analysis excluding the six patients with an unhealthy appendiceal base, overall complications remained similar between groups (19/138 vs. 5/47; *p* = 0.63), with comparable rates of relevant complications (1 vs. 1; *p* = 0.46) (Table 2).

**Table 2:** Comparison of complications between double and single endoloop groups

Complications	Double Endoloop (*n* = 141)	Single Endoloop (*n* = 50)	*p*-value
Overall complications	20 (14.1%; 95% CI 8.4–19.9)	8 (16.0%, 95% CI 5.8–26.2)	0.92
Relevant complications	14 (9.9%, 95% CI 5.0–14.8)	6 (12.0%, 95% CI 3.0–21.0)	0.07
Overall complications (after excluding unhealthy base)	19	5	0.63
Relevant complications (after excluding unhealthy base)	1	1	0.46

Data are presented as number (percentage) with 95% confidence intervals. Sensitivity analyses are shown as counts only due to small event numbers

### Histological findings

Histopathology confirmed acute appendicitis in 166 cases (87%), chronic appendicitis in 3 (2%), normal appendix in 10 (5%), lymphoid hyperplasia in 8 (4%), and other pathology in 4 cases (2%), which included neoplasm and diverticulitis of appendix.

### Qualitative findings

A focus group discussion was conducted with eight surgeons from the emergency general surgery unit at our district general hospital. It explored factors influencing the choice between single and double-endoloop ligation during laparoscopic appendicectomy. Participants included three consultant surgeons and five surgical trainees; one Senior House Officer (SHO), two specialty trainees year 3 (ST3), and two specialty trainees year 4 (ST4). Participant demographics are summarised in Table 3.

**Table 3:** Participant demographics

Participants	*N* = 8
Gender	Male: 6, Female: 2
Training Level	Consultant: 3
	SHO*: 1
	ST3*: 2
	ST4*: 2

*SHO – senior house officer, first 1–2 years of surgical training in the UK

*ST3/ST4 – specialty trainee year 3 and 4 respectively

Thematic analysis identified three key themes:

#### Theme 1: perceived safety and tradition

Surgeons frequently associated the use of two endoloops with a greater sense of security, despite acknowledging a lack of evidence to support superiority. Double-ligation was often described as habitual rather than evidence-based, influenced by surgical culture and training tradition; “I use two because this is how I learnt it 20 years ago” one consultant noted. Perceived security was a large influence amongst trainees; an ST4 trainee explained, “If one endoloop were to fail then you’ve got a backup. I’m sure there’s probably no evidence to it; it just feels more secure.” Participants described their continued use of two endoloops as “out of dogma,” reflecting a persistence of traditional training practices over evidence-based adaptation.

#### Theme 2: assessment of appendiceal base integrity

Intraoperative context was an important determinant of surgical technique. Surgeons reported tailoring their approach to the health and tissue condition of the appendiceal base. There was agreement amongst the group that an unhealthy appendiceal base may not always accommodate two endoloops. One senior consultant said: “It depends on how healthy the base is — you can’t put two endoloops on a necrotic base”. Other surgeons reported using alternative ligation methods altogether, like clips or staplers, in such cases.

#### Theme 3: training opportunities

Training needs also played a role in the decision to apply one or two endoloops. One consultant explained: “The second loop is for practice, for training, when the registrar or the SHO is doing it.” A second ligation was sometimes used to facilitate trainee involvement in stump closure, often used when trainees were still developing confidence in the procedure, with the first endoloop applied by a more experienced surgeon and the second placed by the trainee for practice.

## Discussion

In this single-centre retrospective cohort study comparing single versus double endoloop ligation in laparoscopic appendicectomy, no statistically significant differences were detected in 30-day post-operative complication rates. The incidence of intra-abdominal collection and stump-related sequelae was low in both groups, and no stump leaks were identified. Complication rates were comparable between both groups (9.9% vs. 12%), with cases requiring image-guided drainage remaining low (2.8% vs. 4%). These findings are consistent with existing comparative studies, which similarly report no clear clinical advantage of double ligation [5, 11]. Celik et al. (2019) found no reduction in complications but observed longer operative times in the double-loop group [11], while Rudnicki et al. (2023) reported no stump leaks and a 1% intra-abdominal abscess rate, irrespective of closure method [5]. Evidence from randomised and comparative studies likewise shows that additional loops are associated with longer operating time without improvement in postoperative safety. Taken together, stump integrity and quality of ligature placement appear more important than the number of loops applied. Given the rarity of stump-related complications, this study is unlikely to be sufficiently powered to detect rare but clinically significant differences, and future prospective studies with larger sample sizes are warranted.

Operative time was approximately 24 min shorter in cases where a single endoloop was used (*p* = 0.004). While some increase in operative time with a second loop is expected, particularly when performed by a trainee, the magnitude of this difference is unlikely to be explained solely by loop application time, which experienced surgeons estimated at approximately seven minutes [9]. Differences in case complexity, surgeon seniority, and trainee involvement are therefore likely contributors. Nevertheless, in the context of emergency operating lists, even modest reductions in operative time may improve list efficiency and optimise peri-operative resource use.

The qualitative findings provide important context for understanding why variation in stump closure practice persists despite similar clinical outcomes. Three key themes emerged: perceived safety and tradition, assessment of appendiceal base integrity, and training opportunities. Surgeons frequently described double ligation as a habitual or culturally reinforced practice, reflecting long-standing training patterns rather than evidence-based benefit. This mirrors wider surgical behaviour in which tradition and perceived security may outweigh empirical data. Conversely, others emphasised the influence of intra-operative findings, particularly opting for a single endoloop or an alternative ligation method when the appendiceal base tissue appeared unhealthy. The use of an additional loop as a training opportunity further highlights the educational dynamics that shape operative decisions. These findings underscore that stump closure is a nuanced clinical judgement influenced by pathology, experience, training needs, and institutional culture rather than a binary technical choice.

This study has limitations. Its retrospective single-centre design, modest sample size, and the potential under-reporting of complications managed at other hospital trusts limit generalisability. The unequal distribution of patients between the single- and double-endoloop groups reflects real-world practice patterns but may also limit statistical power and precision of between-group comparisons. The higher proportion of perforated appendicitis in the single-loop group introduces potential confounding that could not be adjusted for within the available dataset. The study is also underpowered to detect rare outcomes such as stump leak, and intra-abdominal collections may result from underlying perforation rather than ligature failure. Furthermore, laparoscopic appendicectomy is inherently variable, influenced by surgeon experience, trainee participation, patient anatomy, and the degree of appendiceal inflammation, all of which may affect both operative time and stump closure strategy.

Despite these limitations, integrating clinical outcomes with surgeon perspectives provides a more holistic understanding of why practice varies. While quantitative findings align with existing literature, the qualitative component provides novel insight into training culture, perceived security, and intra-operative decision-making in a contemporary UK setting. Multi-centre prospective studies are warranted to refine selection criteria and support evidence-based standardisation of practice.

## Conclusions

In this mixed-methods study of laparoscopic appendicectomy, single endoloop stump ligation was associated with similar rates of clinically relevant complications and shorter operative time compared with double ligation. However, given the retrospective design, imbalance in disease severity, and limited statistical power for rare events, these findings should not be interpreted as evidence of equivalence. Surgeons’ use of two endoloops was largely driven by tradition, perceived security, and training opportunities rather than demonstrated clinical advantage. Larger prospective multicentre studies incorporating severity adjustment and cost analysis are warranted to refine selection criteria and support standardisation of practice.

## Supplementary Information

Supplementary Material 1.

## Data Availability

The datasets generated and analysed during the current study are not publicly available due to patient confidentiality and institutional data-protection regulations. An anonymised version of the dataset analysed during the current study is available from the corresponding author on reasonable request and subject to approval by London North West University Healthcare NHS Trust.

## Abbreviations

1EL: Single endoloop ligation
2EL: Double endoloop ligation
ASA: American Society of Anesthesiologists
HCRW: Health and Care Research Wales
HRA: Health Research Authority
IAA: Intra-abdominal abscess
LA: Laparoscopic appendicectomy
REC: Research Ethics Committee
SPSS: Statistical Package for the Social Sciences
SSI: Surgical site infection
