# Supplementary material for: Single or double endoloop ligation in laparoscopic appendicectomy: a mixed-methods study of clinical outcomes and surgeon perspectives
Source: BMC Surg. 2026 Feb 2;26:169. doi: 10.1186/s12893-026-03544-5 (PMC12952101; doi:10.1186/s12893-026-03544-5)
Supplement: Supplementary file 1 — Supplementary Material 1. [file 12893_2026_3544_MOESM1_ESM.docx]

**Appendix 1**

Northwick Park Hospital,

Watford Road,

Harrow, Middlesex.

HA1 3UJ

**PARTICIPANT INFORMATION SHEET**

**Single vs Double Endoloop in Laparoscopic Appendicectomy:**

**A Retrospective Analysis and Surgeon Perspectives.**

1. **Invitation paragraph**

You are being invited to take part in a research study. Before you decide it is important for you to understand why the research is being done and what it will involve. Please take time to read the following information carefully and discuss it with others if you wish. Ask us if there is anything that is not clear or if you would like more information. Take time to decide whether or not you wish to take part.

1. **What is the purpose of the study?**

The purpose of the study is to explore surgeon perspectives on when to use endoloops to secure the base of the appendix in a laparoscopic appendicectomy and specifically when and why they may use one compared to two endoloops.

1. **Rationale for the study**

Despite laparoscopic appendicectomy (LA) being the preferred approach for managing acute appendicitis, variability remains in how surgeons secure the appendiceal stump. Endoloops are widely used due to their cost-effectiveness, but no consensus exists on whether one or two endoloops provides better outcomes.

Limited research suggests no significant difference in complications, though a single endoloop may reduce costs and operative time. Given the importance of operative efficiency and resource allocation in surgical practice, further investigation into the benefits of using one versus two endoloops is warranted. Additionally, there is limited qualitative data on surgeon decision-making regarding endoloop use. Understanding the factors influencing their choice, such as case complexity, surgeon experience or institutional preferences, could provide valuable insights into standardising best practices.

1. **Why have I been chosen?**

You have been selected to take part in the study because you are a general surgery registrar or consultant with extensive experience in performing a laparoscopic appendicectomy.

1. **Do I have to take part?**

It is up to you to decide whether or not to take part. If you do decide to take part you will be given this information sheet to keep and be asked to sign a consent form. If you decide to take part you are still free to withdraw at any time and without giving a reason. A decision to withdraw at any time, or a decision not to take part, will not affect you or your legal rights in any way.

6. **What will happen to me if I take part?**

If you do decide to participate in the study, you will partake in a focus group discussion regarding endoloop use in laparoscopic appendicectomy which will take place on MS Teams for approximately 60 minutes on an agreed date and time.

The discussion will be recorded and transcribed on MS Teams. Once the transcript is generated and anonymised, the original recording will be permanently deleted.

All data collected about you will be anonymised and not shared with anyone. You will have no further involvement in the study and you clinical or medical care will not be affected.

You can withdraw from the study at any point without giving any reason and your medical care or legal rights will not be affected. If you decide to withdraw from the study the data already collected from you will be kept. All information entered onto the database will be anonymised and you will not be identifiable.

8. **What are the possible disadvantages and risks of taking part?**

There are no anticipated risks or disadvantages of taking part.

9. **What are the possible benefits of taking part?**

There are no benefits of taking part in this study, but your contribution would be a valuable aid in research aiming to improve patient outcomes after laparoscopic appendicectomy.

11. **What if something goes wrong?**

If you are harmed by taking part in this research project, there are no special compensation arrangements. If you are harmed due to someone’s negligence, then you may have grounds for a legal action but you may have to pay for it. Regardless of this, if you wish to complain, or have any concerns about any aspect of the way you have been approached or treated during the course of this study, the normal National Health Service complaints mechanisms should be available to you. You should first contact the research team. You may contact our PALS service on 020 8869 5118.

12. **Will my taking part in this study be kept confidential?**

**How will we use information about you?**

We will need to use information from you for this research project. This information will include your name and contact details. People will use this information to do the research or to check your records to make sure that the research is being done properly.

People who do not need to know who you are will not be able to see your name or contact details. Your data will have a code number instead.

**London North West Healthcare NHS Trust** is the sponsor of this research.

**London North West Healthcare NHS Trust** is responsible for looking after your information.

We will keep all information about you safe and secure by:

- Storing the audio-visual recording of the MS Teams focus group discussion in a secure NHS cloud on OneDrive. This will be transcribed and anonymised and the original recording immediately deleted.
- Anonymising the transcript will involve removing identifiable information. You will be given a unique study number which will be used instead of your name in the transcript. The identifier key will be stored separately to the role profiles and interview transcripts.
- Anonymised quotes from the focus group discussion may be used in the study. Your job role or level of experience may be used to characterise the quotes, but no identifiable information will be used.

**International transfers**

Your data will not be shared outside the UK.

**How will we use information about you after the study ends?**

Once we have finished the study, we will keep some of the data so we can check the results. We will write our reports in a way that no-one can work out that you took part in the study.

We will keep your study data for a maximum of 5 years. The study data will then be fully anonymised and securely archived or destroyed.

**What are your choices about how your information is used?**

- you can stop being part of the study at any time, without giving a reason, but we will keep information about you that we already have
- you have the right to ask us to access, remove, change or delete data we hold about you for the purposes of the study. You can also object to our processing of your data. We might not always be able to do this if it means we cannot use your data to do the research. If so, we will tell you why we cannot do this

**Where can you find out more about how your information is used?**

You can find out more about how we use your information:

- our leaflet [www.hra.nhs.uk/patientdataandresearch](http://www.hra.nhs.uk/patientdataandresearch)
- by asking one of the research team

13. **What will happen to the results of the research study?**

The results from the research will be published in scientific journals, but you will not be identified in any publications

14. **Who is organising and funding the research?**

The Chief Investigator of the study is consultant surgeon Mr Jasim Al-Musawi and the study coordinator is core surgical trainee Dr Lara Nassar.

We are not being provided any funding for this study and no one from the research team will be paid for doing the study.

15. **Who has reviewed the study?**

The study has been reviewed by the local Research and Innovation Department at LNWUH.

16. **Contact for Further Information**

For further information you may contact Dr Lara Nassar at [lara.nassar1@nhs.net](mailto:lara.nassar1@nhs.net)

***A copy of the Patient Information Sheet will be given to the participant and a copy kept in the research files.***

**Appendix 2**

IRAS ID: 355719

Centre Number:

Study Number:

Participant Identification Number for this trial:

**CONSENT FORM**

Title of Project: Single vs Double Endoloop in Laparoscopic Appendicectomy

Name of Researcher:

Please initial box

1. I confirm that I have read the information sheet dated 18.05.2025 (version 1.0) for the
   above study. I have had the opportunity to consider the information, ask questions and have
   had these answered satisfactorily.
2. I understand that my participation is voluntary and that I am free to withdraw at any time
   without giving any reason, without my medical care or legal rights being affected.
3. I understand that the information collected about me will be used to support
   other research in the future and may be shared anonymously with other researchers.
4. I agree to take part in the above study.

Name of Participant Date Signature

Name of Person Date Signature

seeking consent

**Appendix 3**

**Semi-Structured Focus Group Interview Questions**

Start recording and transcription on MS Teams.

Confirm consent verbally.

Questions/ Discussion points:

1. What methods of appendix base ligation do you routinely use?
2. In which cases do you use endoloop?
3. Is there any situations where you would not use endoloops?
4. Do you routinely use one or two endoloops? And why?

These questions will be used as a guide, allowing for flexibility and in-depth discussion.
